# mlstverse-web: advancing real-time identification of mycobacteria from sputum using targeted sequencing NALC-Seq

**DOI:** 10.1007/s10142-025-01609-x

**Published:** 2025-05-21

**Authors:** Yuki Matsumoto, Kiyoharu Fukushima, Shunran Zhang, Yuzy Fauzyah, Daisuke Motooka, Haruko Saito, June Yamauchi, Tadayoshi Nitta, Takuro Nii, Takanori Matsuki, Kazuyuki Tsujino, Keisuke Miki, Hiroshi Kida, Shota Nakamura

**Affiliations:** 1 Department of Infection Metagenomics, Bioinformatics Center, Research Institute for Microbial Diseases (RIMD), Osaka University, Suita, Osaka Japan; 2 Department of Clinical Laboratory, National Hospital Organization, Osaka Toneyama Medical Center, Toyonaka, Osaka Japan; 3 Department of Respiratory Medicine and Clinical Immunology, Osaka University Graduate School of Medicine, Suita, Osaka Japan; 4 Department of Respiratory Medicine, National Hospital Organization, Osaka Toneyama Medical Center, Toyonaka, Osaka Japan; 5 Integrated Frontier Research for Medical Science Division, Institute for Open and Transdisciplinary Research Initiatives, Osaka University, Suita, Osaka Japan; 6 Center for Infectious Disease Education and Research, Osaka University, Suita, Osaka Japan

**Keywords:** Mycobacteria, Species identification, Real-time diagnostics, Targeted capture sequencing, Drug resistance prediction

## Abstract

The identification of mycobacteria, including Mycobacterium tuberculosis (MTB) and non-tuberculous mycobacteria (NTM), remains a critical challenge in clinical and public health settings due to their pathogenicity and increasing drug resistance. Traditional diagnostic methods, such as PCR and mass spectrometry, are limited by species detectability and prolonged culture requirements. To address these limitations, this study introduces a novel approach, named NALC-Seq, which integrates next-generation sequencing (NGS) and target capture sequencing for the direct and comprehensive identification of mycobacteria from sputum samples. A total of 54 patients were prospectively or retrospectively enrolled between April 6, 2021, and November 8, 2022, and sputum samples were subjected to next generation sequencing. The NALC-Seq methodology utilizes custom-designed RNA probes for targeted enrichment of mycobacterial DNA, coupled with species identification via the mlstverse-web system. This system integrates web, upload, head, and cloud nodes to streamline data management and analysis. Our evaluation demonstrated that NALC-Seq achieved high sensitivity (98.1%) and accuracy (83.3%) in detecting diverse mycobacterial species, including rare and drug-resistant subspecies. There were discrepancies with MGIT-Seq in nine samples. These samples exhibited low smear positivity rates, and the detection of environmental mycobacteria suggested potential contamination. Furthermore, real-time identification using the MinION device significantly reduced the turnaround time from 686 to 19 h.These findings highlight the potential of NALC-Seq and mlstverse-web as an integrated diagnostic solution for overcoming the limitations of current methods. The proposed approach offers a rapid, accurate, and comprehensive strategy for mycobacterial identification, with significant implications for clinical diagnostics and public health surveillance.

**Supplementary Information:**

The online version contains supplementary material available at 10.1007/s10142-025-01609-x.

## Background

Mycobacteria constitute a substantial genus comprising over 200 species, including *Mycobacterium tuberculosis* (MTB), the causative agent of tuberculosis (TB), *Mycobacterium leprae*, the causative agent of leprosy, and non-tuberculous mycobacteria (NTM). TB ranks among the top three infectious diseases globally, with an estimated 10.6 million cases reported (Pai et al. 2023). These mycobacterial species exhibit pathogenic potential and frequently display resistance to multiple drugs, necessitating varied treatment strategies. Multidrug- or rifampicin-resistant MTB has afflicted approximately 450,000 individuals in 2021, primarily in developing countries (Vanino et al. 2023; Prasad et al. 2014). Conversely, in developed nations, the incidences of NTM diseases are increasing whereas the number of TB cases is declining (Falkinham 2016; Chin et al. 2020). NTM strains demonstrate a broad antimicrobial resistance, with significant variation among subspecies, notably the *Mycobacterium abscessus* complex, which exhibits high-level multidrug resistance (Saxena et al. 2021; Johansen et al. 2020; Shahraki et al. 2015). In contrast, Mycobacterium avium complex (MAC), one of the most frequently encountered NTM species, often exhibits multidrug resistance, posing a significant challenge in clinical treatment (Busatto et al. 2019). Misdiagnosis of NTM diseases has been reported in regions with high TB prevalence (Saptawati et al. 2019; Maiga et al. 2012). Infections caused by multidrug-resistant MTB and emerging NTM strains pose significant global health challenges (Adjemian et al. 2017; Prevots and Marras 2015; Namkoong et al. 2016).

Comprehensive identification of mycobacterial species is time-consuming due to the limited testing methods covering the full spectrum of mycobacterial diversity, often creating bottlenecks in treatment. While PCR-based methodologies are commonly employed for mycobacterial species identification, their accuracy is constrained within a limited species range (Avaniss-Aghajani et al. 1996; Hyeyoung et al. 2000; Horne et al. 2019). Mass spectrometry offers comprehensive identification potential but is limited to species level accuracy and requires bacterial culture, extending the process by up to eight weeks (Alcolea-Medina et al. 2019; Claydon et al. 1996). Although next-generation sequencing (NGS) allows accurate identification at the subspecies level and facilitates phenotype estimation, it suffers from prolonged culture periods akin to conventional methods. Proof of concept studies utilizing NGS for rapid mycobacterial identification have reported same-day identification, particularly focusing on tuberculosis from sputum samples (Votintseva et al. 2017). Alternatively, highly sensitive identification methods have been proposed using RNA probes targeting mycobacteria, enabling major mycobacterial identification from sputum and cultures using a Mycobacteria Growth Indicator Tube (MGIT) (Kambli et al. 2021; He et al. 2020; Mann et al. 2023). Despite the existence of some proof-of-concept studies, routine mycobacterial identification still relies on bacterial culture, which limits the practical application of direct sputum sequencing. Furthermore, the spectrum of mycobacterial species identifiable through direct sequencing remains limited in comparison to the over 200 recognized species within the mycobacterial genus.

In a previous study, we developed the core system of mlstverse, a method for comprehensive mycobacterial species identification using NGS (Matsumoto et al. 2019). We successfully reduced the identification period to approximately 14 d from MGIT samples (Fukushima et al. 2023). However, the widespread adoption of NGS-based identification methods faces barriers, including a demand for bioinformatics expertise. The skill set required for handling NGS data differs from that of clinical tests conducted by laboratory technicians, necessitating bioinformaticians. Although these issues are being addressed through the development of various bioinformatics tools (Yang et al. 2022; Hunt et al. 2019; Florensa et al. 2022; Feldgarden et al. 2019; Sheka et al. 2021), conventional analysis pipelines require the initiation of analysis after sequence completion, resulting in delays. Unlike short-read sequencing, the clinical application of Nanopore sequencing provides sequential output of reads and the potential for shorter turnaround times, making it promising for rapid diagnostics in infectious diseases (Lang 2022; Zheng et al. 2023). In this study, we adapted our identification method to the MinION device, enabling real-time sequence analysis and immediate result visualization through a web interface. We also established a target capture sequencing-based method designed for mycobacteria, facilitating genomic DNA enrichment in sputum and enabling direct sequencing without culture. Using this platform, we demonstrated comprehensive mycobacterial identification at the subspecies level within 24 h.

## Methods

### System overview of mlstverse-web

**Fig. 1:**
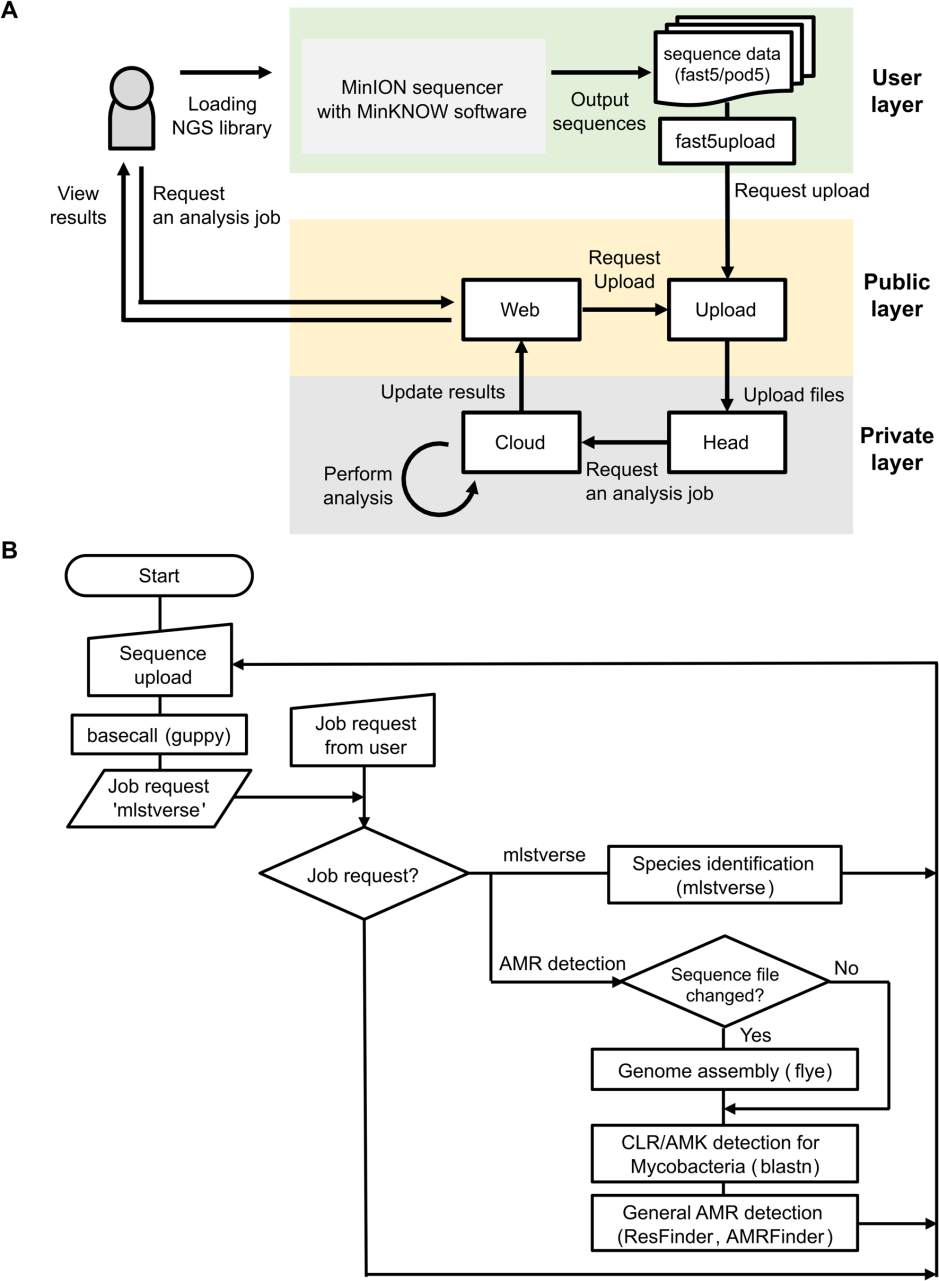
System overview. **A** System components: The system comprises *Web*, *Upload*, *Head*, and *Cloud* nodes. **B** Analysis pipeline for species identification and antimicrobial resistance (AMR) detection

**Fig. 2:**
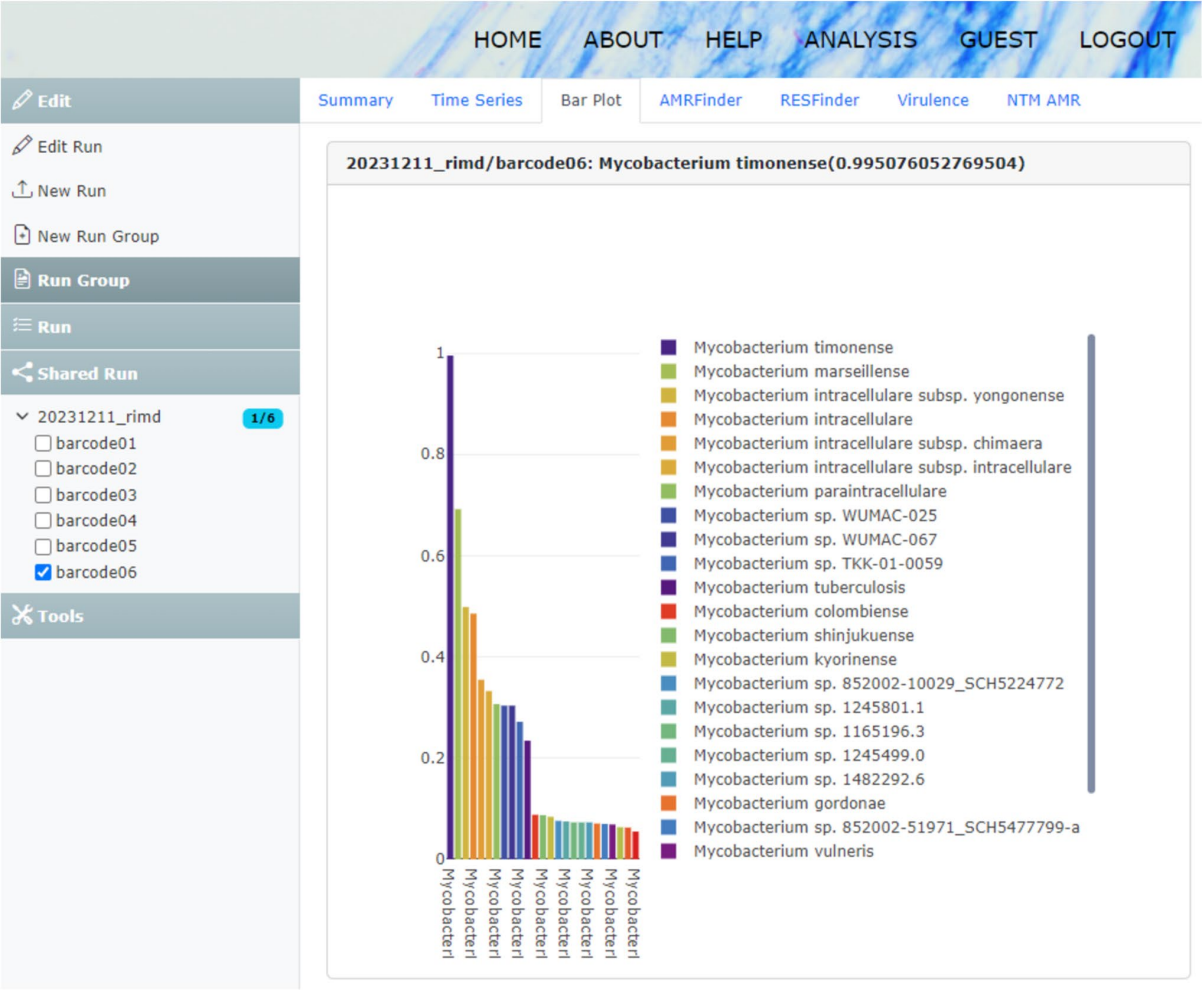
Result overview on mlstverse-web

The mlstverse-web system comprises four key components (Fig. 1A): *Web* nodes, serving as the front end for analysis and result visualization; *Upload* nodes, operating on a host connected to the MinION sequencer and facilitating the transfer of raw signal outputs during sequencing to cloud storage; *Head* nodes, responsible for managing file exchanges and job management; and *Cloud* nodes, which execute actual analysis processing in the cloud. Initially, upon commencing sequencing on the MinION device, the *Upload* node transfers the raw sequence files to cloud storage. The *Head* nodes monitor these file updates and, upon detection of an update, initiate a new analysis job through slurm, an open source job management system (Yoo et al. 2003). Once the sequencing files are uploaded, the analysis jobs are automatically executed on the cloud nodes. The analysis pipeline is illustrated in Fig. 1B. Given the prevalence of multidrug resistance in pulmonary mycobacterial infections, in addition to species identification, the system conducts drug resistance prediction through a web interface. This includes evaluating genetic markers such as *rrs*, *rrl*, and *erm*(*41*) to predict resistance to key drugs such as clarithromycin and amikacin. Moreover, assessments are conducted utilizing tools such as ResFinder and AMRFinder software (Fukushima et al. 2023; Florensa et al. 2022; Feldgarden et al. 2019). Results are accessible via the *Web* node using a web browser, facilitating functional analyses, re-analyses, and results downloads. The *Web* node is publicly accessible at https://mlstverse.org for all users (Fig. 2).

### Clinical sample collection, bacterial culture and MIC testing

A total of 54 patients were enrolled, including individuals previously diagnosed with relatively rare mycobacterial species. This cohort comprised 21 males and 23 females with an average age of 72.48 (± 13.63) years, as detailed in Supplementary Table 2. Sputum samples were prospectively collected between April 6, 2021, and May 27, 2021. Five of these samples were used for the evaluation of mycobacteria quantity via metagenomic shotgun sequencing, and 39 samples were used for NALC-Seq evaluation. In addition, 15 samples containing strains less frequently encountered in the prospective collection, such as MTB, rapidly growing mycobacteria (RGM), *Mycobacterium kansasii*, and *Mycobacterium lentiflavum*, were retrospectively selected from specimens collected between June 22, 2022, and November 8, 2022, and used for NALC-Seq evaluation. Sputum samples obtained from patients underwent preprocessing with the sputum agglutination procedure using Sputazyme (Kyokuto Pharmaceutical Industrial Co., Ltd., Tokyo, Japan) at room temperature for 15 min, followed by centrifugation and supernatant removal. The resulting pellet then underwent N-acetyl-L-cysteine-sodium hydroxide (NALC-NaOH) treatment using MycoPrep (Becton Dickinson, BD, USA) according to the manufacturer's protocol for 15 min, after which the supernatant was discarded. The processed material was used for conventional culture, MGIT culture, and DNA extraction. Conventional culture involved inoculation onto 2% Ogawa medium and incubation at 36 °C for up to 8 weeks. MGIT cultures were performed using the Bactec MGIT 960 system (BD) for up to 6 weeks. MIC testing for clarithromycin and amikacin was performed as previously described (Fukushima et al. 2023).

### DNA extraction and library preparation

Genomic DNA was extracted from residual sputum and Mycobacteria Growth Indicator Tube (MGIT) cultures using the bead shaking method. Briefly, 250 µL of bacterial solution underwent mechanical disruption with 0.1 mm diameter glass beads (MN Bead Tubes Type B, cat# 740,812.50, Takara Bio, Shiga, Japan), using a Magnalyzer device for 45 s at a speed of 4500 rpm. Following mechanical disruption, the bead tubes were centrifuged at 13,000 rpm for 5 min, followed by heat treatment at 95 °C for 5 min using a block incubator. Subsequently, 100 µL of the supernatant was collected.

The mycobacterial gene panel was designed using SureDesign (https://earray.chem.agilent.com/suredesign) to cover the complete loci set of the MLST database of mlstverse. On average, 150 ng of extracted DNA was sheared using the Covaris M220 focused-ultrasonicator (Covaris, Woburn, MA, USA) to 600 bp. Sequence libraries comprising 54 samples were prepared using a custom SureSelect Low Input Target Enrichment System (Agilent Technologies Inc. Santa Clara, CA, USA), following the manufacturer’s instructions, and sequenced using MGI DNBSEQ-G400 (MGI, Shenzhen, PRC) with paired-end 100 bp reads. The sequencing reads were analyzed and identified using mlstverse. For fifteen samples newly collected in this study, MGIT-Seq was performed and results were compared with the targeted capture sequencing. The sequencing reads were analyzed and identified using the mlstverse. For the 15 samples newly collected in this study, MGIT-Seq was conducted, and the results were subsequently compared with those derived from targeted capture sequencing.

For real-time sequencing analysis, the library was prepared from the amplicon produced in the enrichment step of the 2nd PCR using the Ligation Sequencing Kit (SQK-LSK109, Oxford Nanopore Technologies (ONT), Oxford, UK) with Native Barcoding Expansion 1–12 (EXP-NBD104, ONT) and sequenced using MinION with flow cell R9.4.1 (FLO-MIN106D, ONT).

### Data analysis and pipeline

Raw output data obtained from MinION sequencing was basecalled using guppy software on high accuracy mode. Long reads (> 1000 bp) with an average quality score below Q10 were filtered, while short reads from MiSeq and DNBSeq-G400 had adapter sequences and regions below Q30 trimmed. For mlstverse analysis, long and short sequence reads were mapped to MLST loci sequence file using the minimap2 (Li 2018) and bwa-mem (Li 2013), respectively. The MLST score, ranging from 0 to 1 according to the proportion of detected loci, was calculated based on mapping results using mlstverse (Matsumoto et al. 2019). Species were identified based on the profile with the highest score, and profiles with scores below 0.1 were excluded. Genome assembly was conducted using the entire fastq file using flye (Kolmogorov et al. 2019). Clarithromycin and amikacin resistance were predicted by searching the sequences of the *rrs*, *rrl* and *erm(41)* genes using blastn (Camacho et al. 2009) and evaluated the following method described in Fukushima et al. (2023). Resistance mutations A2058G/A2059G (*rrl*) and A1408G (*rrs*) were evaluated. For erm(41), gene presence, truncations, and the T28 C mutation were assessed. General drug resistance detection was conducted using ResFinder (Florensa et al. 2022) and AMRFinder (Feldgarden et al. 2019).

### Metagenomic sequencing analysis of sputum mycobacteria

A sequencing library was prepared from the genomic DNA derived from the five sputum samples using Nextera XT DNA Library Preparation Kit (Illumina, San Diego, CA, USA) according to the manufacturer’s instructions. The sequencing run was performed using the MiSeq Reagent Kit v2 (300 Cycles, Illumina). The taxonomic compositions were evaluated using Kraken2 (Wood et al. 2019) and species identification was performed using mlstverse (Matsumoto et al. 2019).

### Evaluation of target capture sequencing for mycobacterial detection

*Mycobacterium agri* JCM 6377 was cultured in Middlebrook 7H9 medium with OADC Enrichment (BD-BBL) at 37 °C with 150 rpm on a shaker incubator. Genomic DNA was extracted from the bacterial pellet using Power Soil Pro according to the manufacturer's instructions. The input gDNA was prepared for each 10 ng of human gDNA, adding 1, 10, 100, 1000, or 10,000 pg of *M. agri* gDNA, respectively. Sequence libraries were prepared using a custom SureSelect Low Input Target Enrichment System (Agilent Technologies Inc. Santa Clara, CA, USA), according to the manufacturer’s instructions, and sequenced using MGI DNBSEQ-G400 (MGI, Shenzhen, PRC) with paired-end 100 bp reads.

### Benchmarking of captured sputum sequences

Input DNA for benchmarking sensitivity and specificity of capture sequencing was prepared by mixing the gDNA of humans, Microbial Community DNA Standard (ZymoBIOMICS, D6305), and *M. agri* in the ratio of 1:0:0 (Human), 0:1:0 (Mock), 0:0:1 (*M. agri*), 1:0:1 (A50_H50), 0:1:1 (A50_M50), and 0:9:1 (A10_M90) respectively. Sequencing libraries were prepared in the same manner as others using custom SureSelect libraries.

### Real-time mycobacterial detection of clinical sputum samples

A sequencing library was prepared from the genomic DNA derived from the sputum sample #54 utilizing custom SureSelect libraries for real-time sequencing analysis. The sequencing output of MinION was automatically uploaded to the mlstverse web server by the fast5upload program (https://github.com/ymatsumoto/fast5upload). The fast5 files were basecalled using guppy basecaller. Species identification analysis was performed using mlstverse v0.2.1 and the mlstverse.Mycobacterium.db database. All analyses were automatically submitted from the head node via the job management system and executed on the cloud node.

## Results and discussion

### Presence of mycobacterium in sputum metagenome

**Fig. 3:**
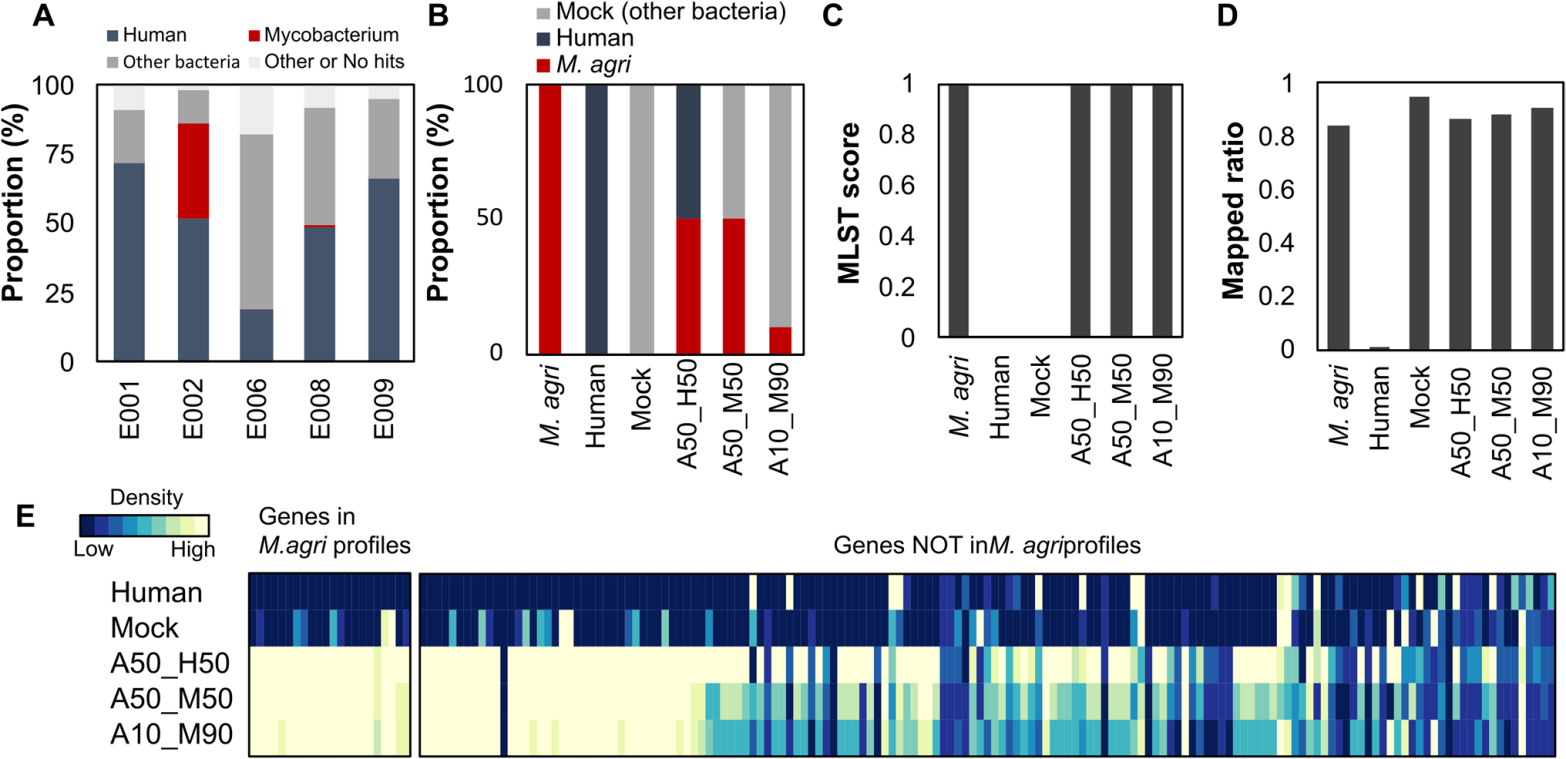
Evaluation of sputum metagenome and NALC-Seq. **A**. Taxonomic compositions resulting from metagenomic sequencing. **B**. Artificial mock compositions for target capture sequencing. **C**. MLST score identified as *M. agri*. **D**. Ratio of mapped reads in total reads. **E**. Density of reads mapped in each loci

Clinical samples such as sputum typically comprise over 50% of DNA derived from the human genome or oral bacteria (Votintseva et al. 2017; Doughty et al. 2014). Nicholls et al. demonstrated that achieving adequate sequencing depth is challenging when the amount of target DNA in the metagenome is very low, even with the use of whole genome amplification (Nicholls et al. 2019). Prior to evaluating target capture sequencing, we assessed the sputum metagenome for the presence of mycobacteria using metagenomic shotgun sequencing. We sequenced five clinical sputum samples that tested positive for smear tests at a grade of 1 + or higher. Among these reads, those originating from the *Mycobacterium* genus ranged from 0.1% to 34%, with an average of 7.0% (Fig. 3A). Leveraging this sequencing data, we conducted an MLST analysis. Only one sample, which exhibited the highest proportion of mycobacterium (34%), was successfully identified as *Mycobacterium avium* subsp. *hominissuis*, with an MLST score of 0.83 (Supplementary Table 1). As noted in previous studies (Votintseva et al. 2017; Doughty et al. 2014), we observed a very low abundance of mycobacteria in sputum. These findings were consistent with prior research from other investigators (Votintseva et al. 2017; Doughty et al. 2014), underscoring the necessity for genomic enrichment to obtain sufficient DNA from sputum samples.

### Assessment of target capture sequencing for mycobacterial detection

To assess the efficacy of RNA probes in detecting mycobacteria from sputum samples, we prepared simple DNA mixtures comprising target (*M. agri*) and non-target (human) components. Subsequently, we conducted target capture sequencing to evaluate the sensitivity of the RNA probes. The proportion of reads mapped from the captured DNA ranged from a maximum of 97% with a 10 ng (10,000 pg) target to a minimum of 3% with a 1 pg target (Table 1). Notably, even with a low mapped read ratio of 0.10 from a 1 pg target, the MLST score reached a value of 1.00 (Table 2). Moreover, probe concentration did not significantly impact capture efficiency, even up to a tenfold dilution (0.1X) of the manufacturer-suggested concentration.

**Table 1:** Mapped read ratio. Mapped read ratio in each condition when sequencing a total of 10 ng of mixed DNA comprising human and *M. agri* DNA. Each column of the table indicates the quantity of *M. agri* DNA

Amount of probes (X)	*M. agri* gDNA (pg)				
	1	10	100	1000	10,000
0.01	0.03	0.11	0.50	0.90	0.92
0.03	0.04	0.32	0.75	0.95	0.93
0.1	0.09	0.43	0.88	0.94	0.96
0.3	0.13	0.61	0.92	0.98	0.97
1	0.10	0.50	0.88	0.97	0.97

**Table 2:** MLST score using mlstverse

Amount of probes (X)	*M. agri* gDNA (pg)				
	1	10	100	1000	10,000
0.01	0.85	0.99	1.00	1.00	1.00
0.03	0.97	1.00	1.00	1.00	1.00
0.1	0.99	1.00	1.00	1.00	1.00
0.3	1.00	1.00	1.00	1.00	1.00
1	1.00	1.00	1.00	1.00	1.00

Subsequent specificity assessment involved six conditions of DNA mixtures (Fig. 3B), comprising target (*M. agri*) and non-target (human or commercial bacterial mock). In conditions where the input DNA included *M. agri* (specifically, in conditions labeled as *M. agri*, A50_H50, A50_M50, and A10_M90), all 24 loci present within the *M. agri* profiles were successfully detected, achieving MLST scores of 1.00 (Fig. 3C). Conversely, no identification scores were obtained in conditions lacking *M. agri* (labeled as Human and Mock), despite enrichment and alignment of some sequences to the database (illustrated in Figs. 3D and E).

The observed increase in non-target sequences alongside a decrease in target DNA is likely attributable to residual DNA in the tube during the bead purification process, a phenomenon known as carryover (Jennings et al. 2017). The estimated amount of carryover DNA is approximately 10 pg, as evidenced by the proportion of mapped sequences reaching 50% at a genomic DNA (gDNA) quantity of 10 pg. Thus, even from 1 pg of target within 10 ng of non-target DNA, approximately 10% of target sequences can be obtained, enabling successful species identification. This detection threshold corresponds to a cell concentration of approximately 2.9 × 10^5^ cells/mL, equivalent to a G2 on the Gaffky scale and a smear-positive of 1 + (Okada 1949). The designed RNA probe library facilitates highly sensitive mycobacterial detection, with an extremely low likelihood of false positives from non-mycobacterial DNA sources present in sputum samples. Subsequently, we coined this methodology NALC-Seq, after the NALC-NaOH treatment conducted prior to library preparation.

### Comprehensive mycobacterial detection via target capture sequencing

We conducted the target capture sequencing, termed NALC-Seq, followed by identification analysis using mlstverse and compared the identification results with those of culture isolates. The identification results of culture isolates were as follows: twenty-one identified as *M. avium*, thirteen as *M. intracellulare*, five as *M. abscessus* subsp*. massiliense*, three as *M. abscessus* subsp*. abscessus/bolletii*, four as *M. tuberculosis*, four as *M. kansasii,* and one each as* Mycobacterium paragordonae, Mycobacterium mucogenicum/phocaicum, Mycobacterium fortuitum* complex*,* and *M. lentiflavum* (Supplementary Table 3). Among these patients, 51 cases (98.1%) were identified as positive for either MTB or NTM.

**Fig. 4:**
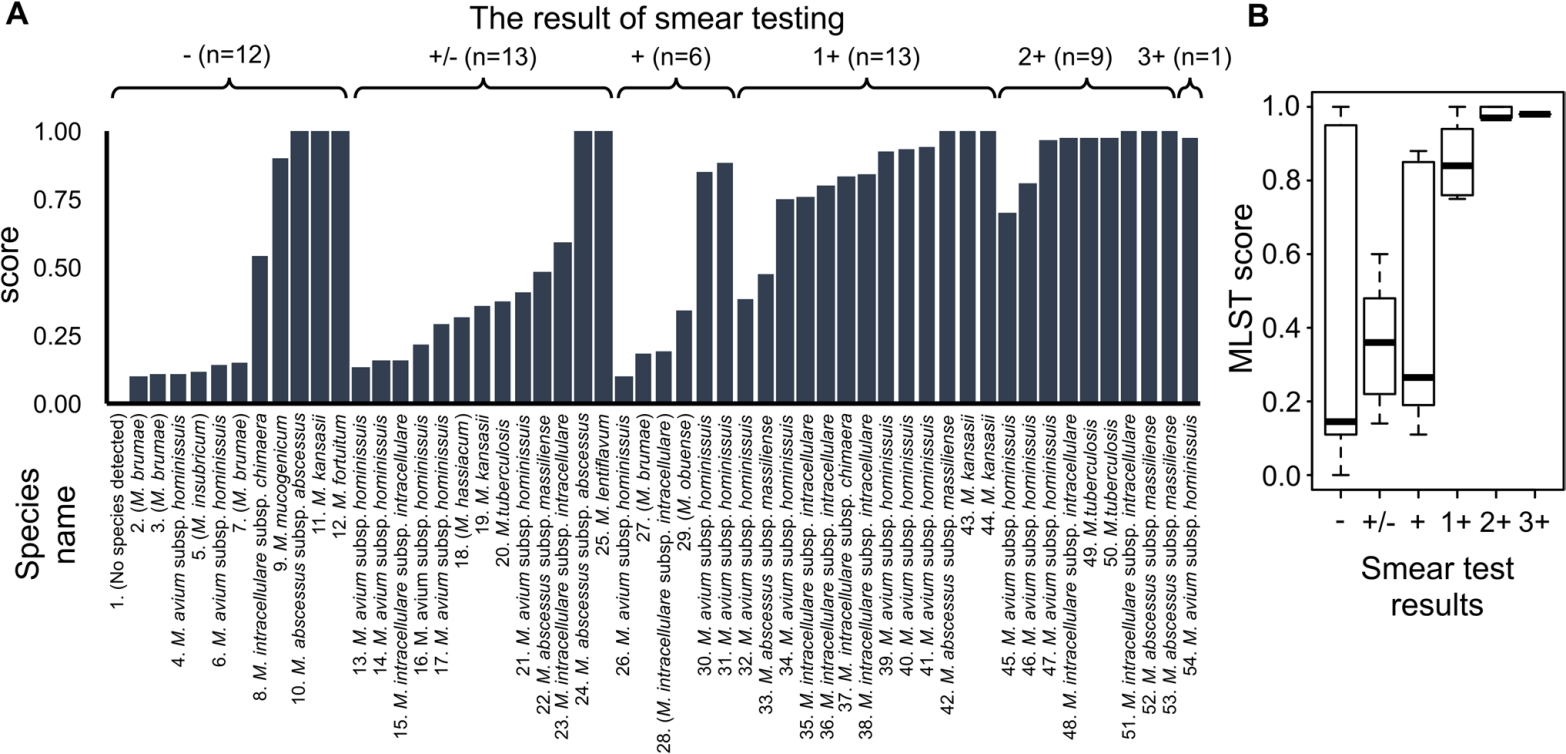
Comprehensive species identification of mycobacteria from sputum samples using NALC-Seq. **A**. Top score in MLST analysis and identified species name. species names enclosed in parentheses represent inconsistent results with the MGIT-Seq. **B**. Boxplot of MLST score by Smear-Positive level

DNA extracted from 54 sputum samples underwent sequencing using NALC-Seq. Across all 54 samples, NALC-Seq demonstrated a sensitivity of 98.1%, yielding MLST scores in 53 out of the 54 samples (Fig. 4A and Table 3). Consistent identification results were observed in 45 out of 54 samples compared to culture isolates, resulting in an accuracy of 83.3% (Fukushima et al. 2023) (Supplementary Tables 3 and 4). Among the samples, nine exhibited discrepancies with MGIT-Seq results, being identified as *Mycobacterium brumae* and *Mycobacterium obuense* typically found in environmental sources such as water and soil, indicating possible environmental mycobacteria detection in the samples of lower smear positivity. In cases with strong smear positivity (1 + or higher), sensitivity and accuracy achieved 100.0%, with an average MLST score exceeding 0.75 (Figs. 4A and B).

**Table 3:**
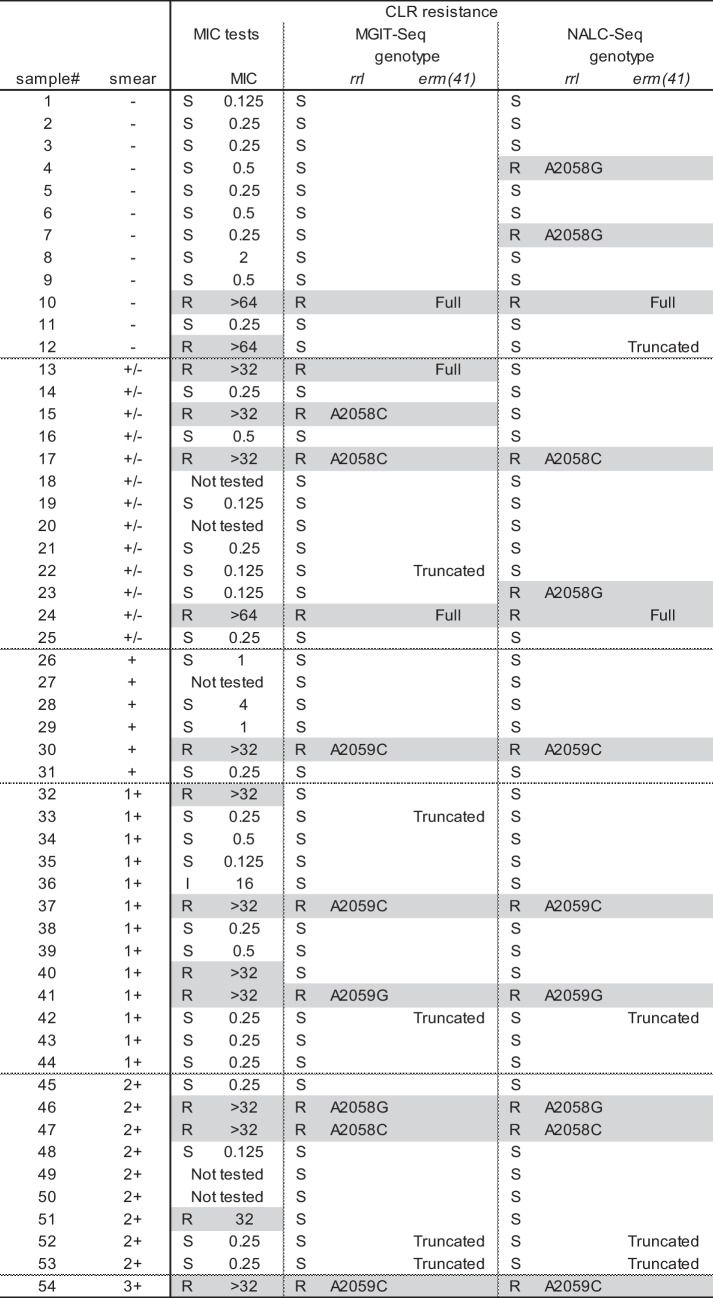
Comparison with conventional methods

Compared to drug susceptibility testing results for 49 isolates, discrepancies in resistance prediction were observed for 4 isolates with MGIT-Seq and 9 isolates with NALC-Seq; furthermore, there were discrepancies between MGIT-Seq and NALC-Seq results for 5 isolates. Among the discrepancies between MGIT-Seq and NALC-Seq, 2 isolates (#12, #15) were false negatives only with NALC-Seq. For the remaining 3 isolates (#4, #7, #23), false-positive clarithromycin resistance was detected by NALC-Seq from smear-negative samples. Notably, 2 of these 3 isolates with low smear grades exhibited low scores, suggesting potential false-positive detection due to sequences originating from pulmonary and oral bacteria. The diagnostic validities of macrolide resistance prediction using NALC-Seq were as follows: sensitivity, 0.80; specificity, 0.912; positive predictive value, 0.80; and negative predictive value, 0.912. Regarding amikacin resistance, while resistance (MIC 64 ug/mL) was observed only in a single isolate (#46), the prediction based on sequencing data was negative, resulting in a discrepancy.

It is commonly acknowledged that the sensitivity of direct mycobacterial detection from sputum using PCR is approximately 80% (Noordhoek et al. 1994; Li et al. 2017; Anand and Biswas 2021; Sarro et al. 2021). Sarro et al. demonstrated the capability to detect MTB and common NTM, achieving a sensitivity of 83.3% and a specificity of 96.6% (Sarro et al. 2021) using multiplex PCR. He et al. conducted capture sequencing similar to our approach, achieving a sensitivity of 91.3% and an accuracy of 83.3% for 30 samples, including MTB and 32 common pathogenic NTM (He et al. 2020). These results indicate that NALC-Seq offers sensitivity and accuracy comparable to traditional direct identification methods from sputum. Additionally, the custom probes for NALC-Seq are designed based on the sequences of 186 mycobacterial species (Supplementary Table 5), significantly surpassing conventional methods (Matsumoto et al. 2019). The method successfully identified relatively rare species such as *M. abscessus*, *M. lentiflavum*, *M. mucogenicum*, *M. fortuitum*, and *M. kansasii* among the patients enrolled in this study. Based on these results, we established NALC-Seq as a method for comprehensive identification of a diverse range of mycobacterial species using a standardized approach.

### Real-time identification using NALC-Seq and mlstverse-web

**Fig. 5:**
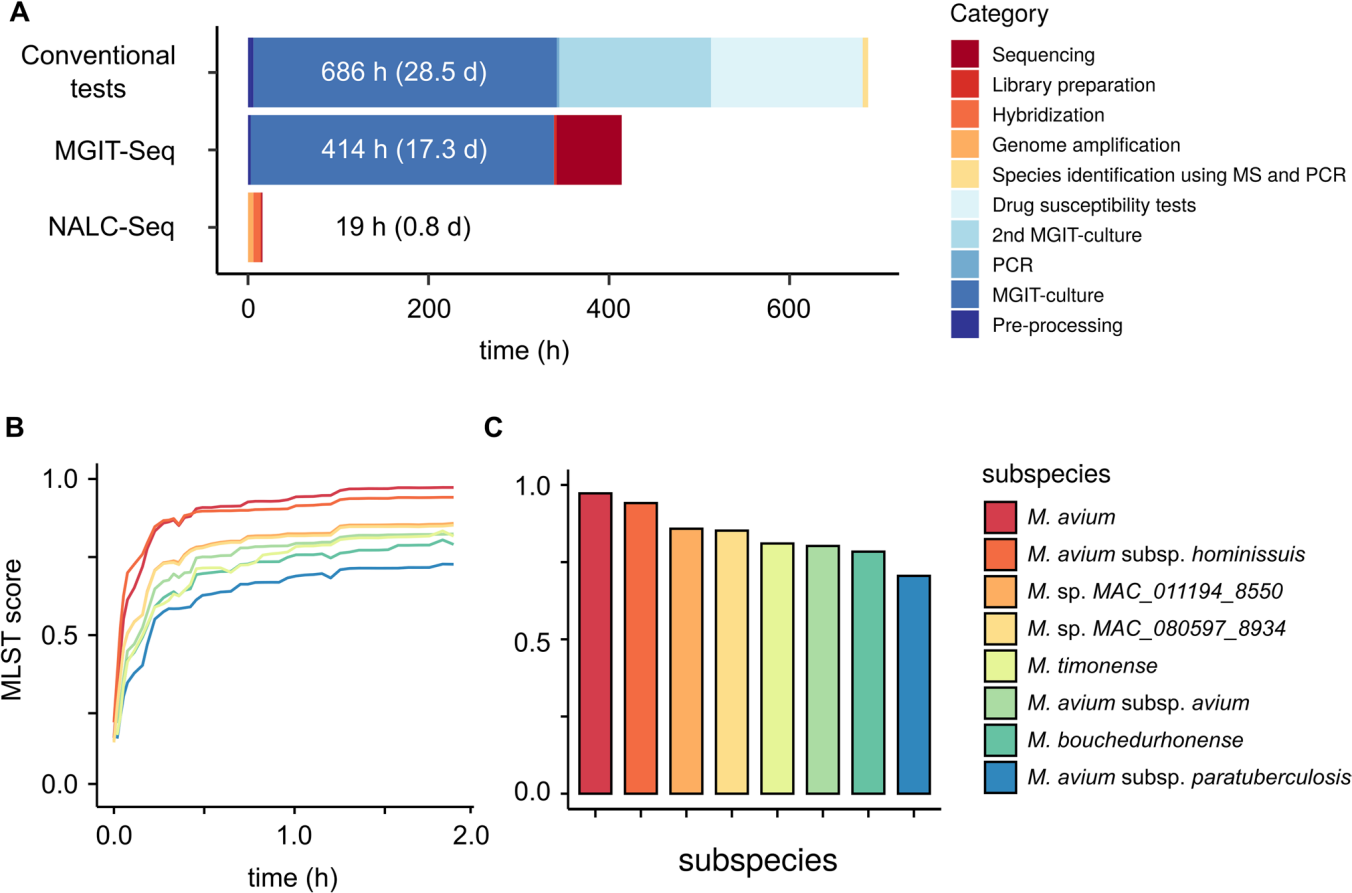
Experimental flow and comparison of time required for NALC-Seq. **A**. Estimated duration in library preparation. **B**. Time course of MLST score. **C**. MLST score 2 h from sequencing start

To expedite species identification and achieve faster diagnosis, we demonstrated the integration of NALC-Seq with the MinION device along with a real-time analysis pipeline. We re-sequenced the genomic DNA of sample #54 (depicted in Fig. 4), previously identified as *M. avium* subsp. *hominissuis*, using the MinION and mlstverse-web system. Within 2 h of sequencing initiation, we obtained consistent identification results with the highest MLST score (Fig. 5A). The time series analysis of MLST scores (Fig. 5B) revealed that the score for *M. avium* subsp. *hominissuis* surpassed those of other species by over 50% within approximately 10 min and stabilized within 30 min. The total processing time for this real-time NALC-Seq analysis was 0.8 d (19 h), marking a significant reduction compared to conventional testing, which typically takes 28.5 d (686 h), and MGIT-Seq, which requires 17.3 d (414 h) (Fig. 5C) (Fukushima et al. 2023). NALC-Seq notably broadened the range of identifiable bacterial species, effectively overcoming limitations associated with existing capture sequencing and direct sequencing methods (Votintseva et al. 2017; Kambli et al. 2021; He et al. 2020; Mann et al. 2023). Additionally, using mlstverse-web system is not exclusive to NALC-Seq and can be seamlessly employed with just a MinION device and a laptop computer, eliminating the need for specialized bioinformatics expertise. This demonstration represents a substantial advancement over traditional methods, significantly reducing the time required for species identification and diagnosis from weeks to mere hours, through the seamless integration of NALC-Seq with the MinION platform and a real-time analysis pipeline.

The limitations of this study include the relatively small number of samples evaluated, the cost disadvantage compared to high-throughput short-read sequencers, the limited number of evaluated pretreatment methods, the limited types of detectable drug resistance, and the potential inability to detect species beyond the 186 mycobacterial species used to design the custom probes listed in Supplementary Table 5.

## Conclusion

In conclusion, the development of NALC-Seq represents a significant advancement in the diagnostics of mycobacterial infection. This study introduces a novel approach for the rapid and comprehensive identification of mycobacteria directly from sputum samples, effectively surpassing the limitations of traditional diagnostic methods. By integrating target capture sequencing with the mlstverse-web system and enabling real-time analysis on the MinION device, this research addresses the urgent need for expedited diagnostic methods amid escalating drug resistance and the global health burden associated with mycobacterial diseases. Our findings underscored the efficacy of NALC-Seq in identifying a wide range of mycobacterial species from smear-positive sputum samples, including rare and drug-resistant strains, with remarkable sensitivity and accuracy. This methodology not only streamlines treatment decisions by providing rapid and precise diagnostic information but also holds promise for significantly impacting public health through enhanced disease surveillance and control. Future research should prioritize further optimizing this technology to simplify its operation and broaden its applicability to a wide range of infectious agents, thus contributing to the global effort to combat infectious diseases.

## Supplementary Information

Below is the link to the electronic supplementary material.

Supplementary file1 (XLSX 9 KB)

Supplementary file2 (XLSX 10 KB)

Supplementary file3 (XLSX 16 KB)

Supplementary file4 (XLSX 39 KB)

Supplementary file5 (XLSX 17 KB)

## Data Availability

The website of mlstverse is available at https://mlstverse.org. An automated uploader for raw sequencing data is publicly available on github (https://github.com/ymatsumoto/fast5upload). Sequencing data used in this study is deposited in National Center for Biotechnology Information with the accession PRJDB12894 and PRJNA1077745.
